# Engineered antibody Fc variant with selectively enhanced FcγRIIb binding over both FcγRIIa^R131^ and FcγRIIa^H131^

**DOI:** 10.1093/protein/gzt022

**Published:** 2013-06-05

**Authors:** F. Mimoto, H. Katada, S. Kadono, T. Igawa, T. Kuramochi, M. Muraoka, Y. Wada, K. Haraya, T. Miyazaki, K. Hattori

**Affiliations:** Research Division, Chugai Pharmaceutical Co., Ltd., Gotemba, Shizuoka, Japan; Research Division, Chugai Pharmaceutical Co., Ltd., Gotemba, Shizuoka, Japan; Research Division, Chugai Pharmaceutical Co., Ltd., Gotemba, Shizuoka, Japan; Research Division, Chugai Pharmaceutical Co., Ltd., Gotemba, Shizuoka, Japan; Research Division, Chugai Pharmaceutical Co., Ltd., Gotemba, Shizuoka, Japan; Research Division, Chugai Pharmaceutical Co., Ltd., Gotemba, Shizuoka, Japan; Research Division, Chugai Pharmaceutical Co., Ltd., Gotemba, Shizuoka, Japan; Research Division, Chugai Pharmaceutical Co., Ltd., Gotemba, Shizuoka, Japan; Research Division, Chugai Pharmaceutical Co., Ltd., Gotemba, Shizuoka, Japan; Research Division, Chugai Pharmaceutical Co., Ltd., Gotemba, Shizuoka, Japan

**Keywords:** antibody engineering, FcγRIIb, Fc engineering, inhibitory FcγR, platelet activation

## Abstract

Engaging inhibitory FcγRIIb by Fc region has been recently reported to be an attractive approach for improving the efficacy of antibody therapeutics. However, the previously reported S267E/L328F variant with enhanced binding affinity to FcγRIIb, also enhances binding affinity to FcγRIIa^R131^ allotype to a similar degree because FcγRIIb and FcγRIIa^R131^ are structurally similar. In this study, we applied comprehensive mutagenesis and structure-guided design based on the crystal structure of the Fc/FcγRIIb complex to identify a novel Fc variant with selectively enhanced FcγRIIb binding over both FcγRIIa^R131^ and FcγRIIa^H131^. This novel variant has more than 200-fold stronger binding affinity to FcγRIIb than wild-type IgG1, while binding affinity to FcγRIIa^R131^ and FcγRIIa^H131^ is comparable with or lower than wild-type IgG1. This selectivity was achieved by conformational change of the C_H_2 domain by mutating Pro to Asp at position 238. Fc variant with increased binding to both FcγRIIb and FcγRIIa induced platelet aggregation and activation in an immune complex form *in vitro* while our novel variant did not. When applied to agonistic anti-CD137 IgG1 antibody, our variant greatly enhanced the agonistic activity. Thus, the selective enhancement of FcγRIIb binding achieved by our Fc variant provides a novel tool for improving the efficacy of antibody therapeutics.

## Introduction

IgG-based monoclonal antibodies (mAbs) have become important therapeutic options for numerous diseases (Brekke and Sandlie, 2003; Maggon, 2007). Fcγ receptors (FcγR)-dependent cytotoxicity plays an important role in the antitumor activity of mAbs for cancer immunotherapy (Nimmerjahn and Ravetch, 2012).

Several works describe engineering the Fc region to enhance the effector function of mAbs by increasing the binding affinity for active FcγRs (FcγRIa, FcγRIIa and FcγRIIIa) with amino acid substitutions. For example, antibodies engineered to have increased binding affinity for FcγRIIIa exhibited superior *in vitro* ADCC activity and *in vivo* antitumor activity compared with wild-type mAbs (Stavenhagen *et al.*, 2007; Zalevsky *et al.*, 2009). In addition to protein engineering, glyco-engineered tumor-specific mAbs with afucosylated N-linked oligosaccharides at Asn297 in the Fc region showed increased binding affinity for human FcγRIIIa and mouse FcγRIV, which resulted in enhancing the therapeutic activity in mouse models (Nimmerjahn and Ravetch, 2005; Mossner *et al.*, 2010).

In contrast to these activating FcγRs that function as immunostimulatory receptors, inhibitory FcγRIIb is reported to function as an immunomodulatory receptor (Li and Ravetch, 2011; White *et al.*, 2011). The inhibitory receptor FcγRIIb is the only IgG Fc receptor expressed on B-cells and plays a critical role in regulating B-cell homeostasis (Heyman, 2003; Nimmerjahn and Ravetch, 2008). Immune complexes (ICs) coengage FcγRIIb and B-cell receptor (BCR) and then selectively suppress B-cells that recognize cognate antigen (Heyman, 2003). FcγRIIb also regulates other B-cell stimulators that amplify B-cell proliferation and differentiation and suppresses the expression of costimulatory molecules (Leibson, 2004; Crowley *et al.*, 2009).

FcγRIIb also plays an inhibitory role in a homeostatic checkpoint of dendritic cells (DCs) for inducing tolerance or immunity by ICs, in contrast to FcγRIIa. While ligation of FcγRIIa led to DC maturation, targeting FcγRIIb alone did not activate DCs (Boruchov *et al.*, 2005). FcγRIIb on DCs induced peripheral tolerance by inhibiting antigenic processing and DC activation to suppress T-cell activation (Desai *et al.*, 2007).

Besides these inhibitory effects of FcγRIIb, several groups have recently reported that FcγRIIb enhances the agonistic activity of anti-tumor necrosis factor receptor (TNFR) superfamily antibody by working as a scaffold (White *et al.*, 2013). Proliferation of antigen-specific T-cells induced by anti-CD40 antibodies was abrogated in FcγRIIb-deficient mice (White *et al.*, 2011; Li and Ravetch, 2012). Anti-death receptor 5 (DR5) agonist antibody also required FcγRIIb to exert its agonistic activity and initiate apoptosis in lymphoma cells (Wilson *et al.*, 2011). The FcγRIIb-expressing cells are considered to work as a scaffold to efficiently crosslink anti-CD40 or DR5 antibodies bound to target cells via FcγRIIb.

In order to exploit these properties, engineering Fc region to increase the binding affinity to FcγRIIb is considered to be a promising approach. Introducing S267E/L328F substitutions into the Fc region of human IgG1 increased the binding affinity to FcγRIIb 430-fold without increasing that to FcγRI, FcγRIIa^H131^ or FcγRIIIa (Chu *et al.*, 2008). Anti-CD19 antibody with the Fc promoted suppression of B-cell activation and proliferation in SLE mouse model by coengaging FcγRIIb with BCR (Horton *et al.*, 2011). Recent reports noted that anti-IgE antibody with S267E/L328F Fc variant reduced the production of IgE *in vivo* (Chu *et al.*, 2012) and that the Fcɛ domain fused with S267E/L328F Fc variant suppressed degranulation and calcium mobilization of mast cells (Cemerski *et al.*, 2012). Moreover, agonistic antibodies against CD40 or DR5 showed more potent agonistic activity *in vivo* with enhanced FcγRIIb binding (Li and Ravetch, 2011, 2012). These reports clearly demonstrate that engineered Fc with enhanced binding to FcγRIIb has various therapeutic applications. However, it was reported that S267E/L328F substitutions also enhanced the binding to one of the FcγRIIa allotypes, FcγRIIa^R131^, to a level similar to the binding to FcγRIIb (Smith *et al.*, 2012). Therefore, when applying the substitutions to mAb therapeutics, the consequence of increasing the binding to FcγRIIa should be considered.

It is reported that a high incidence of thromboembolic complication was observed in patients treated with anti-CD154 or anti-VEGF antibody in clinical settings (Boumpas *et al.*, 2003; Scappaticci *et al.*, 2007). The ICs composing of these antibodies and the antigens activated platelets and induced thrombosis in human FcγRIIa transgenic mice by crosslinking FcγRIIa expressed on the platelets (Meyer *et al.*, 2009; Robles-Carrillo *et al.*, 2010). Moreover, intravenous immunoglobulins also induced FcγRIIa-mediated platelet aggregation *in vitro* (Pollreisz *et al.*, 2008). These results demonstrate that even wild-type IgG1 generally has a potential risk of inducing thromboembolism through an FcγRIIa-dependent mechanism, suggesting that this risk could be increased if the binding to FcγRIIa is enhanced. Therefore, considering the therapeutic applications, antibody with selectively enhanced binding to FcγRIIb relative to both FcγRIIa^R131^ and FcγRIIa^H131^ is preferred.

In this work, we investigated antibodies with selectively enhanced binding to FcγRIIb over both FcγRIIa^R131^ and FcγRIIa^H131^. We screened a large set of single substituted variants of human IgG1 for binding to human FcγRs and found a distinct variant that distinguishes FcγRIIb from both FcγRIIa allotypes. We solved its crystal structure in complex with human FcγRIIb and elucidated the structural recognition mechanism by which it recognizes FcγRIIb over both FcγRIIa allotypes. In order to improve its FcγRIIb binding, we conducted further optimization and identified a novel antibody variant with over 200-fold more enhanced binding to FcγRIIb without increasing the binding to either FcγRIIa allotype. Structural analysis of this variant revealed the mechanism of the improved binding to FcγRIIb. We also report that antibody that also has enhanced binding to FcγRIIa is more likely to activate platelets and induce aggregation and would have a shorter *in vivo* half-life compared to wild-type IgG1. We also confirmed that this variant enhanced the agonistic activity of anti-CD137 antibody *in vitro*. Our results indicate that the novel Fc variant with purely selectively enhanced binding to FcγRIIb is applicable to a broad range of therapeutic antibodies, since it avoids increasing the potential to activate or aggregate platelets and to have rapid clearance *in vivo*.

## Materials and methods

### Preparation of IgG1 variants

IgG1 variants used in the experiments were expressed transiently in FreeStyle™ 293F cells transfected with plasmids encoding heavy and light chain and purified from culture supernatants using protein A. Site-directed mutagenesis of the Fc region was performed using QuikChange Site-Directed Mutagenesis Kit (Stratagene) or In-Fusion HD Cloning Kit (Clontech).

### Construction, expression and purification of FcγRs

The genes encoding the extracellular region of human FcγRs were synthesized based on the sequence information obtained from the National Center for Biotechnology Information. FcγRs were fused with 6× His-tag at their C-terminus. Vectors containing FcγRs were transfected into FreeStyle™ 293F cells. The receptors were purified from the harvested culture supernatants by using ion exchange chromatography, nickel affinity chromatography and size exclusion chromatography.

### Binding and affinity analysis for human FcγRs by surface plasmon resonance (SPR)

The interaction of antibody variants with FcγRs was monitored using Biacore instruments (GE Healthcare), as previously described (Richards *et al.*, 2008). Antibody variants were captured on the CM5 sensor chip (GE Healthcare) on which antigen peptide or protein A/G (Thermo Scientific) was immobilized, followed by injection of FcγRs. The binding of each variant to each FcγR was normalized by the captured amount of antibody on the sensor chip and was expressed as a percentage of that of IgG1. Kinetic analysis was performed by global fitting of binding data with a 1 : 1 Langmuir binding model using Biacore Evaluation Software (GE Healthcare).

### Crystalization, data collection and structure determination of the complex of Fc fragment of human IgG1 with P238D substitution, Fc(P238D) and FcγRIIb

Crystals were obtained by 1 : 1 mixing of protein complex (10 mg/ml) with 0.1 M Bis-Tris pH6.5, 0.2 M ammonium acetate, 2.7% (w/v) d-galactose, 17% PEG3350 in sitting drop vapor diffusion setups at 20°C. For data collection, crystals were flash frozen at 95 K in precipitant solution containing 22.5% ethylene glycol. Diffraction data to 2.6 Å were collected using the Photon Factory beamline BL-1A. Data were processed with Xia2 (Winter, 2010), XDS Package (Kabsch, 2010) and Scala (Evans, 2006). The structure was determined by molecular replacement with PHASER (McCoy *et al.*, 2007) using Fc part of Fc(IgG1)/FcγRIIIa structure (PDB ID: 3SGJ) and FcγRIIb structure (PDB ID: 2FCB) as search models. The asymmetric unit was formed by a single 1 : 1 complex. A model was built with the program Coot (Emsley *et al.*, 2010) and refined with the program REFMAC5 (Murshudov *et al.*, 2011).

### Crystalization, data collection and structure determination of the complex of Fc fragment of V12 variant, Fc(V12) and FcγRIIb

Crystals were obtained by 1 : 1 mixing of protein complex (10 mg/ml) with 0.1 M Bis-Tris pH6.5, 0.2 M potassium phosphate dibasic, 19% PEG3350 in hanging drop vapor diffusion setups at 20°C. For data collection, crystals were flash frozen at 95 K in precipitant solution containing 20% ethylene glycol. Diffraction data to 2.86 Å were collected at the SPring-8 beamline BL-32XU. After data processing and structure determination by molecular replacement using Fc(P238D)/FcγRIIb structure, a model was built with the program Coot and refined with the program REFMAC5. The asymmetric unit was formed by a single 1 : 1 complex.

Data collection and refinement statistics of both crystals are summarized in Supplementary Table S1. All graphical presentations were prepared with PyMOL (DeLano, 2002).

### Preparation of Fc fragment and FcγRIIb complexes

We cloned recombinant Fc fragments corresponding to the heavy chain residues from 216 (EU numbering) to C-terminus for the crystallization. Cys 220 was replaced with Ser so that the free cysteine would not make disulfide bonds. FcγRIIb expressed by FreeStyle™ 293F cells in the presence of the mannosidase I inhibitor, kifunensine, was purified and treated with endoglycosidase F1 fused with glutathione *S*-transferase for deglycosylation as described previously to minimize the heterogeneity of the oligosaccharide (Grueninger-Leitch *et al.*, 1996). The complexes of Fc fragment and the deglycosylated FcγRIIb were prepared by mixing Fc fragment with a little excess of the deglycosylated FcγRIIb, and purified by size exclusion chromatography.

### Thermal shift assay

Melting temperature (*T*_M_) of the C_H_2 region was measured as previously described (He *et al.*, 2010). The SYPRO orange dye (Invitrogen) was added to 0.1 mg/ml protein solutions at a final dilution of 1 : 500. Fluorescence measurements were employed using a real-time polymerase chain reaction instrument, Rotor-Gene Q (QIAGEN). The temperature was increased from 30 to 99°C at a heating rate of 4°C/min.

### Stability study of high concentration antibodies

Antibodies were concentrated to 100 mg/ml in a histidine buffer (20 mM histidine, 150 mM NaCl, pH6.0) by ultrafiltration (Millipore). The solutions were stored at 25°C to assess the stability of the antibodies. High-molecular-weight (HMW) species were analyzed with TSK-GEL G3000SWXL column (TOSOH) by size exclusion high-performance liquid chromatography at initial time point and after 4 weeks. The mobile phase contained 300 mM NaCl in 50 mM phosphate at pH7.0. The percentage of HMW species peak in total protein peak area was calculated using Empower Waters software.

### Binding affinity analysis of antibodies for human FcRn

Recombinant, soluble human FcRn (hFcRn) was expressed in HEK293 cells and prepared as described previously (Dall'Acqua *et al.*, 2006). The interaction of soluble hFcRn was monitored as described previously (Igawa *et al.*, 2010).

### Pharmacokinetic studies of monoclonal antibodies in hFcRn transgenic mouse

Anti-human interleukin-6 receptor (hIL-6R) IgG1 or V12 variant was administered to human FcRn (hFcRn) transgenic mice (B6.mFcRn^−/−^ hFCRN Tg32 B6.Cg-Fcgrt<tm1Dcr> Tg(FCGRT)32Dcr; Jackson Laboratories) by single intravenous injection together with 1 g/kg Sanglopor (CSL Behring K.K.) to mimic endogenous IgG in human. Blood samples were collected. The concentration of each antibody was determined by capture with anti-idiotype antibody (in-house), followed by addition of hIL-6R and biotinylated anti-hIL-6R antibody (R&D Systems) and detected by Streptavidin-Poly HRP80. The half-life was calculated from the plasma concentration–time data using non-compartmental analysis with WinNonlin Professional software (Pharsight).

### Platelet aggregation and activation study using washed platelets

Fresh blood from healthy volunteers whose FcγRIIa genotype is R/R or H/H was anticoagulated with 0.5 ml of 3.8% sodium citric acid–citrate in blood collection tubes. Platelet-rich plasma (PRP) was obtained by centrifuging at 200 *g* for 15 min and then removing the supernatant. PRP was washed in modified Tyrode buffer (137 mM NaCl, 2.7 mM KCl, 12 mM NaHCO_3_, 0.42 mM NaH_2_PO_4_, 2 mM MgCl_2_, 5 mM HEPES, 5.55 mM dextrose, 0.35% bovine serum albumin) with 1.5 U/ml apyrase and resuspended at a concentration of 3 × 10^8^/ml in modified Tyrode buffer. Washed platelets were then incubated with preformed IC for 5 min. The Preformed IC was prepared by mixing the anti-IgE mAb having different Fc variants (200 µg/ml) with its antigen (229 µg/ml), human IgE, at a molar ratio 1 : 1. Five minutes after the incubation, 30 µM ADP was added to induce the first wave of platelet aggregation. Platelet aggregation was measured by an aggregometer (MCM Hema Tracer 712; MC Medical) at 37°C with stirring at 1000 rpm.

### Cells and reagents

CTLL-2 cells (mouse T lymphocyte cell line, No.RCB0637) were provided by the RIKEN BRC through the National Bio-Resource Project of the MEXT, Japan. Raji cells (human Burkitt's lymphoma cell line, ATCC No.CCL-86) were purchased from the American Type Culture Collection. Both cell lines were cultured in RPMI 1640 medium (Nacalai tesque), supplemented with 10% heat-inactivated foetal bovine serum (Bovogen). The culture medium for CTLL-2 was supplemented with 10 ng/ml recombinant mouse interleukin (IL)-2 (PeproTech). The culture medium for Raji cells was supplemented with 10 mM HEPES, 1 mM sodium pyruvate (Nacalai tesque), 4.5 g/l d-glucose, 1.5 g/l sodium bicarbonate (Sigma-Aldrich).

### Flow cytometry analysis of CD137 expression

To analyze mouse CD137 expression on CTLL-2 cells, anti-mouse CD137 clone 1D8 variable region fused with the Fc domain of human IgG1 (Shuford *et al.*, 1997) or human IgG1 isotype control (Alexis, Lausen) was applied. Goat F(ab′)2 anti-human IgG-FITC #732596 (Beckman Coulter) was used as the detection antibody. Cell Lab Quanta SC MPL system (Beckman Coulter) was used for cell acquisition and data analysis was conducted using FlowJo software (Tree Star Inc.).

### Measurement of T-cell activation by anti-CD137 antibody by cytokine production

CTLL-2 cells (2 × 10^5^/mL) and Raji cells (2 × 10^5^/ml) were co-cultured in RPMI 1640 medium supplemented with 10% heat-inactivated foetal bovine serum (Bovogen), 10 ng/ml mouse IL-2 (PeproTech), 10 ng/ml phorbol 12-myristate 13-acetate, 0.5 μg/ml ionomycin (Sigma-Aldrich). The cells were treated with 3 μg/ml anti-CD137 antibodies with different Fc (Fc of human IgG1, S267E/L328F or V12 variant) for 24 h at 37°C, 5% CO_2_. Mouse interferon (IFN)-γ concentration in the cultured medium was determined by ELISA system (PeproTech), according to the manufacturer's protocol.

## Results

### Screening and characterization of variants to selectively enhance FcγRIIb binding by comprehensive mutagenesis

**Fig. 1.**
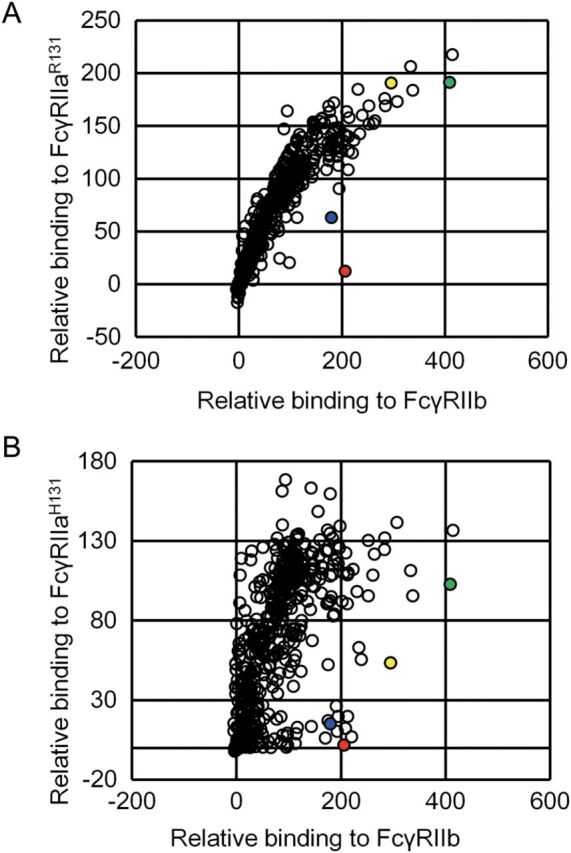
Comprehensive mutagenesis of the lower hinge and C_H_2 region. Binding affinity for FcγRIIa^R131^, FcγRIIa^H131^ and FcγRIIb was evaluated. The binding to FcγRIIa^R131^, FcγRIIa^H131^ or FcγRIIb of template antibody was normalized to 100. The binding to FcγRIIa^R131^ and FcγRIIb is shown in panel **A** and the binding to FcγRIIa^H131^ and FcγRIIb is shown in panel **B**. Novel substitutions to selectively improve FcγRIIb binding, L328E and P238D, are indicated in blue and red, respectively. Previously reported substitutions, S267E and L328F, to improve FcγRIIb but also FcγRIIa^R131^ binding are indicated in green and yellow, respectively.

We screened a large set of over 500 variants prepared by replacing each of about 30 residues in the lower hinge and C_H_2 region with every naturally occurring amino acid except for cysteine, for binding to human FcγRIIa^H131^, FcγRIIa^R131^ and FcγRIIb in order to identify substitutions that selectively enhance FcγRIIb binding relative to both FcγRIIa allotypes. The effect of each substitution on the binding to FcγRIIa^R131^, FcγRIIa^H131^ and FcγRIIb is compared in Fig. 1A and B. Only two substitutions, P238D and L328E, were found to enhance FcγRIIb binding while decreasing the binding to both FcγRIIa allotypes, although previously reported S267E and L328F increased both binding.

**Fig. 2.**
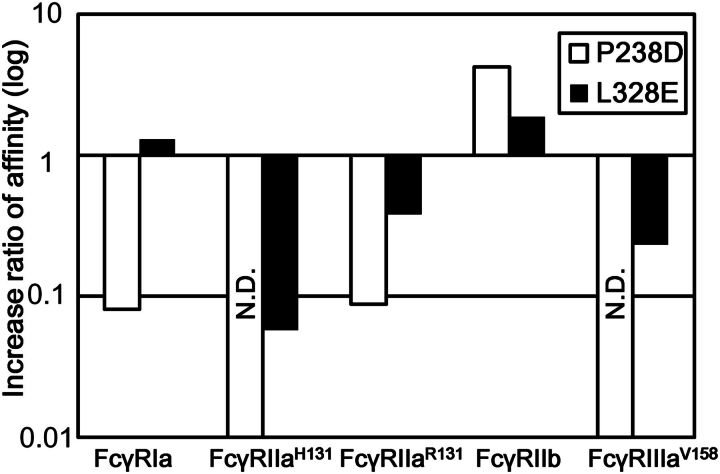
Affinity ratio of Fc variants identified by comprehensive mutagenesis for all the human FcγRs. The binding of P238D variant to FcγRIIa^H131^ and FcγRIIIa^V158^ and that of L328E variant to FcγRIIa^H131^ were not detected. Affinity ratio was calculated by the equation, *K*_D_ (IgG1)/*K*_D_ (Fc variant).

We compared the dissociation constant (*K*_D_) of wild-type human IgG1, P238D variant and L328E variant (Supplementary Table S2) and the changes in affinity from IgG1 are shown in Fig. 2. Both P238D and L328E variants enhanced affinity to FcγRIIb, while reduced affinity to both FcγRIIa^R131^ and FcγRIIa^H131^. P238D variant reduced the affinity for all the active FcγRs and enhanced that for FcγRIIb more significantly than L328E variant.

### Crystal structure of the FcγRIIb complex with IgG1 Fc fragment with P238D

**Fig. 3.**
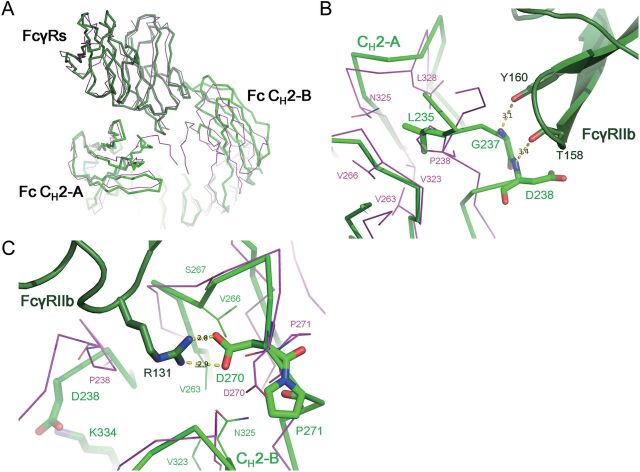
Structural comparison of Fc(P238D)/FcγRIIb complex with Fc(IgG1)/FcγRIIa^R131^ complex. (**A**) The overall structural comparison of Fc(P238D)/FcγRIIb complex with Fc(IgG1)/FcγRIIa^R131^ complex. (**B**) The binding interface between the C_H_2-A domain of Fc(P238D) and FcγRIIb. (**C**) The binding interface between the C_H_2-B domain and FcγRIIb. The structural changes and novel interactions introduced by P238D in both interfaces are shown by comparing Fc(P238D)/FcγRIIb and Fc(IgG1)/ FcγRIIa^R131^. Fc(P238D)/FcγRIIb and Fc(IgG1)/FcγRIIa^R131^ are shown in green and magenta, respectively. FcγRIIb and FcγRIIa^R131^ are shown in the darker color, respectively.

Fc(P238D) bound to FcγRIIb in an asymmetric fashion between the two C_H_2 domains (C_H_-A and C_H_-B) like previously reported Fc(IgG1) and FcγR complexes. In Fig. 3A, the overall structure of Fc(P238D)/FcγRIIb was compared with known Fc(IgG1)/FcγRIIa^R131^ (PDB ID: 3RY6) (Ramsland *et al.*, 2011). Despite the high-sequence homology between FcγRIIb and FcγRIIa^R131^ (Supplementary Fig. S1), the domain arrangement of C_H_2-B in Fc(P238D)-FcγRIIb interface was different from that in Fc(IgG1)/FcγRIIa^R131^.

Structural comparisons of both C_H_2 domains with those of Fc(IgG1) complexed with FcγRIIa^R131^ revealed conformational changes of loops around position 238 in both C_H_2 domains (Fig. 3B and C). Pro238 in wild-type human IgG1 formed an inside hydrophobic core with Val263, Val266, V323, Asn325 and Leu328. On the other hand, Asp238 in P238D variant shifted out of the core and it was exposed to the solvent region. Instead, Leu235 occupied the vacated space which Pro238 occupied in Fc(IgG1), forming another hydrophobic core in C_H_2-A, but not in C_H_2-B. As a result, there was a large cavity surrounded by Val263, Val266, Val273, Val323, Asn325 and Leu328 in C_H_2-B. Asp238 in C_H_2-B formed an electrostatic interaction with Lys334 in the same chain.

In the C_H_2-A domain, the main chain of Gly237 formed a novel hydrogen bond with Tyr160 of FcγRIIb (Fig. 3B). A weak hydrogen bond between the main chain of Asp238 and the side chain of Thr158 of FcγRIIb was also observed. In addition, the side chain of Asp238 formed van der Waals contacts with the side chain of Thr158 in FcγRIIb. In the C_H_2-B domain, the conformation of the loop around Asp270 changed from that of Fc(IgG1) complexed with FcγRIIa^R131^, and Asp270 formed a salt bridge with Arg131 of FcγRIIb (Fig. 3C).

### Screening, characterization and design of variants to further enhance FcγRIIb binding by comprehensive mutagenesis using the P238D variant as a template

In order to further enhance the affinity to FcγRIIb, we combined P238D with L328E or S267E/L328F, previously known variant to increase binding to FcγRIIb and FcγRIIa^R131^. Unexpectedly, the additive effect by combining P238D with those substitutions was not observed. Affinities for FcγRIIb of these variants are listed in Supplementary Table S3.

Then, we screened substitutions to improve FcγRIIb binding when they were combined with P238D by comprehensive mutagenesis using the P238D variant as a template. Approximately 400 variants with an additional single substitution onto the P238D variant were generated, and the affinity for FcγRIIb was determined. Six effective substitutions (E233D, G237D, H268D, P271G, Y296D and A330R) were identified. We further combined these substitutions to create V12 variant having all the six substitutions described above. The fold increases of the variants against FcγRIIb over P238D variant are listed in Table I.

**Table I.** Effect of additional substitution(s) into P238D variant on binding affinity for FcγRIIb

Variant no.	Amino acid change					Fold (*K*_D_ for FcγRIIb)
Single substitution						
V1	E233D					1.7
V2		G237D				1.5
V3			H268D			1.7
V4				P271G		5.0
V5					A330R	1.2
Two substitutions						
V6	E233D				A330R	1.9
Three substitutions						
V7	E233D			P271G	A330R	8.2
V8		G237D	H268D	P271G		9.5
V9		G237D		P271G	A330R	33
Four substitutions						
V10	E233D		H268D	P271G	A330R	9.0
V11		G237D	H268D	P271G	A330R	40
Five substitutions						
V12	E233D	G237D	H268D	P271G	A330R	62

Fold = *K*_D_ (P238D variant)/*K*_D_ (Fc variants).

**Fig. 4.**
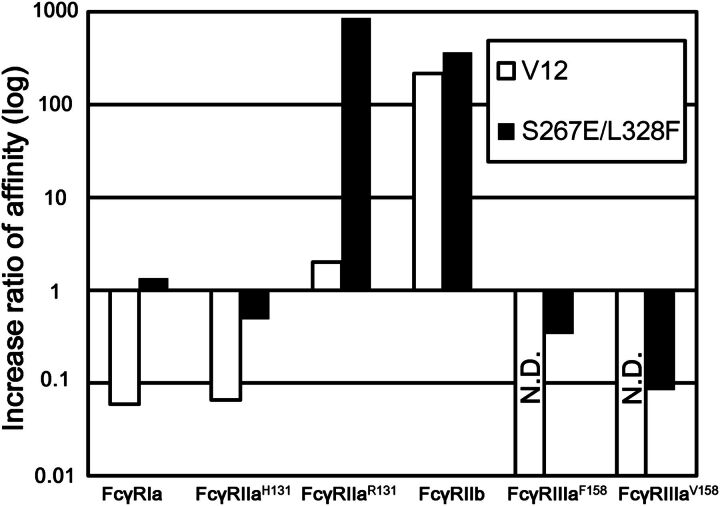
Affinity ratio of S267E/L328F and V12 variant for all the human FcγRs. The binding of V12 variant to FcγRIIIa^F158^ or FcγRIIIa^V158^ was not detected. Affinity ratio was calculated by the equation, *K*_D_ (IgG1)/*K*_D_ (Fc variant).

*K*_D_s of wild-type IgG1, S267E/L328F variant and the most potent variant, V12 variant, to all the FcγRs are listed in Supplementary Table S2 and the changes in affinity from IgG1 are shown in Fig. 4. V12 variant showed 217-fold increase in affinity for FcγRIIb, yet bound to FcγRIIa^R131^ with similar affinity to that of wild-type IgG1 and to the other activating FcγRs with affinity significantly lower than that of IgG1. On the other hand, S267E/L328F variant increased the affinity for FcγRIIb 355-fold and that for FcγRIIa^R131^ even greater, 864-fold.

### Crystal structure of the FcγRIIb complex with Fc(V12)

The same conformational changes around Asp238 induced by P238D substitution in Fc(P238D)/FcγRIIb were also observed in Fc(V12)/FcγRIIb.

**Fig. 5.**
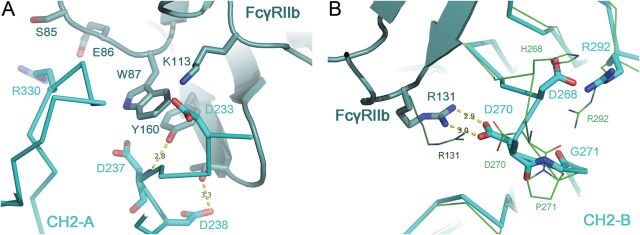
Structure of Fc(V12)/FcγRIIb complex. (**A**) The binding interface between the C_H_2-A domain of Fc(V12) and FcγRIIb. The substitutions in Fc(V12) and the residues in FcγRIIb with a minimum distance of 3.8 Å from these substitutions are shown as sticks. (**B**) The binding interface between the C_H_2-B domain of Fc(V12) and FcγRIIb. The structural changes around Asp270 in the C_H_2-B domains shown by comparing Fc(P238D)/FcγRIIb (green) and Fc(P238D)/FcγRIIb (cyan) complexes. FcγRIIb in each complex is shown in the darker color.

In the C_H_2-A domain, the introduced residues, Asp233, Asp237 and Arg330, were located in the interface with FcγRIIb (Fig. 5A). The weak electron density of Asp233, replaced from Glu, was observed. As Lys113 of FcγRIIb is located near Asp233, they might interact with each other. In the C_H_2-A domain, Asp237, replaced from Gly, made van der Waals contacts with Trp87 and Tyr160 of FcγRIIb. In addition, the hydrogen bond distance between NH of the main chain at position 237 in Fc and OH of the Tyr160 side chain in FcγRIIb changed from 3.1 Å in Fc(P238D) (Fig. 3C) to 2.9 Å in Fc(V12) (Fig. 5A). A weak hydrogen bond between the side chain of Asp238 and the side chain of Thr158 of FcγRIIb was also observed. The electron density of the side chain of Arg330, which replaced Ala, was not clearly observed. As Glu86 of FcγRIIb is located near Arg330, a weak interaction between those residues could be present.

In the C_H_2-B domain, H268D and P271G substitutions induced a further conformational change of a loop around Asp270 (Fig. 5B). The substitution from Pro to Gly at position 271 caused the main chain to flip and consequently rearranged the 266–273 loop and Arg131 of FcγRIIb, which also formed a salt bridge with Asp270 in C_H_2-B of Fc(V12) as well as in Fc(P238D). In addition, the electron density of Gly271 in C_H_2-B was clearly observed, though in Pro271 in Fc(P238D) it was not. This conformational change of the loop caused a rearrangement of Arg292 in C_H_2-B of Fc(V12). The other substituted residue, Asp268, formed an electrostatic interaction with the shifted Arg292.

### Pharmacokinetic and biophysical property assessment of V12 variant

In order to characterize the pharmacokinetic aspect of V12 variant, we measured the affinity for hFcRn and the pharmacokinetics in hFcRn transgenic mice. The binding affinity of V12 variant for hFcRn at pH6.0 was comparable with that of wild-type IgG1 and the *in vivo* half-life was also comparable (Table II).

**Table II.** Characterization of V12 variant

	*T*_M_ (CH2) (°C)	HMW formation after storage (%)	*K*_D_ for FcRn (μM)^a^	Half-life (day)^b^
IgG1	70	0.30	1.5 ± 0.0	12.8 ± 3.8
V12	62	0.33	1.4 ± 0.0	12.6 ± 3.2

The group mean ± SD are given for the parameter (*n* = 3 each).

^a^ *K*_D_ for FcRn was measured at pH6.0 by SPR.

^b^Half-life of intravenously injected IgG1 and V12 variant at 1 mg/kg in hFcRn transgenic mouse.

*T*_M_ of the C_H_2 domain was measured by thermal shift assay. The *T*_M_ of V12 variant decreased by 8°C relative to wild-type IgG1 (Table II). To assess the real-time stability for pharmaceutical application of V12 variant, a stability study at an antibody concentration of 100 mg/ml was performed. The formation of HMW species of V12 variant after storage for 4 weeks at 25°C was comparable with that of wild-type IgG1 (Table II).

### *In vitro* activation and aggregation of platelets by ICs consisting of IgE and anti-IgE antibody with S267E/L328F variant or V12 variant

Platelets obtained from two donors homozygous for FcγRIIa^R131^ genotype and incubated with IC consisting of IgE and anti-IgE S267E/L328F variant increased the expression of CD62p and PAC-1 on the platelets, but those incubated with IC consisting of IgE and anti-IgE with the V12 variant did not (Supplementary Fig. S2A and B). On the other hand, when we used platelets obtained from two donors homozygous for FcγRIIa^H131^ genotype, IC consisting of IgE and anti-IgE S267E/L328F variant slightly upregulated the expression of CD62p and PAC-1 on the platelets but IC consisting of anti-IgE with the V12 variant did not after the incubation compared with the control (phosphate-buffered saline) (Supplementary Fig. S2C and D).

**Fig. 6.**
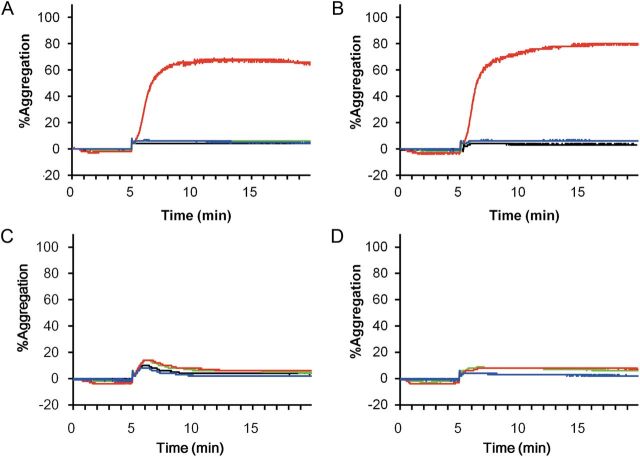
Platelet aggregation studies incubated with ICs. Platelet aggregation was evaluated after platelets were incubated with ICs consisting of IgE with anti-IgE V12 variant (blue), that of anti-IgE S267E/L328F variant (red), IgE and anti-IgE IgG1 (green) or phosphate-buffered saline (black) for 5 min after being primed with ADP. Aggregation of the platelets from two donors with FcγRIIa R/R131 homozygous genotype are shown in panels **A** and **B**, respectively. Aggregation of the platelets from two donors with FcγRIIa H/H131 homozygous genotype are shown in panels **C** and **D**, respectively.

Next, the platelet aggregation induced by ICs was evaluated with an aggregometer. After the addition of ADP, only IC consisting of IgE and anti-IgE with S267E/L328F substitutions aggregated the platelets obtained from two donors homozygous for FcγRIIa^R131^ genotype, while IC consisting of IgE and anti-IgE with V12 variant did not (Fig. 6A and B). On the other hand, IC consisting of neither variant induced the aggregation of the platelets obtained from two donors homozygous for FcγRIIa^H131^ genotype (Fig. 6C and D).

### Enhancement of agonistic activity of anti-CD137 antibody with enhanced FcγRIIb binding Fc

Several reports have described that agonistic anti-TNFR superfamily antibodies generally require FcγRIIb coengagment for their agonistic activity and that enhancing the binding affinity of the antibodies for FcγRIIb could increase the agonistic activity (White *et al.*, 2011; Wilson *et al.*, 2011; Li and Ravetch, 2012). Therefore, we tested whether V12 also has the same property using agonist antibody against CD137 (clone 1D8), which is one of the TNFR superfamily.

**Fig. 7.**
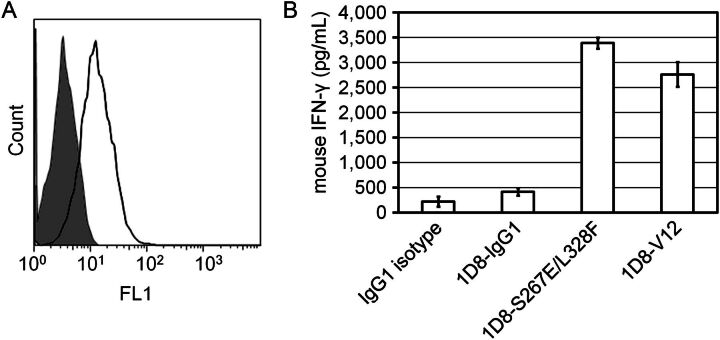
V12 variant enhanced the T-cell activating agonistic activity of anti-CD137 antibody 1D8. (**A**) CD137 surface expression on CTLL-2 cells. Open histogram indicates 1D8-IgG1 and filled histogram indicates IgG1 isotype control. (**B**) T-cell activation induced by anti-CD137 antibody with different Fc (IgG1, V12 variant and S267E/L328F variant) was measured as mouse IFN-γ production of CTLL-2 co-cultured with Raji. Each bar shows mean ± SEM of three independent experiments.

Mouse T lymphoma cell line CTLL-2 was used as mouse CD137-expressing cells (Fig. 7A), and human B lymphoma cell line Raji was used as human FcγRII-positive cells (Hernández *et al.*, 2010). T-cell activating agonistic activity of anti-CD137 antibody was measured with the production of mouse IFN-γ production of CTLL-2 co-cultured with Raji cells. Consistent with the previous reports, V12 variant as well as S267E/L328F variant increased IFN-γ production induced by agonist anti-CD137 antibody compared with intact human IgG1 by more than 5-fold (Fig. 7B).

## Discussion

Several efforts to improve FcγR binding by Fc engineering have been reported to date. While most of the engineering enhances the binding affinity for activating FcγRs (Stavenhagen *et al.*, 2007; Zalevsky *et al.*, 2009; Mossner *et al.*, 2010), engineering to enhance binding affinity to inhibitory FcγR is limited (Chu *et al.*, 2008). S267E/L328F variant, the Fc variant reported to have the highest affinity for inhibitory FcγR binding, enhanced the binding affinity for FcγRIIb by 355-fold; however, it also enhanced the binding affinity for FcγRIIa^R131^ to the same level as FcγRIIb (Smith *et al.*, 2012). The high similarity between FcγRIIb and FcγRIIa^R131^ suggests that discriminating FcγRIIb and FcγRIIa^R131^ by Fc engineering is challenging (Supplementary Fig. S1).

In this work, we investigated the substitutions to distinguish FcγRIIb from FcγRIIa^R131^ by comprehensive mutagenesis and discovered a highly selective substitution, P238D, which provides the highest selectivity for FcγRIIb relative to all the other active FcγRs including FcγRIIa^R131^. Fc variants which discriminate FcγRIIb from FcγRIIa^R131^ were extremely rare, probably because FcγRIIb is highly homologous to FcγRIIa^R131^ (Supplementary Fig. S1) and the Fc-FcγRIIb interface would be also highly homologous to the Fc-FcγRIIa^R131^. To the best of our knowledge, this is the first report illustrating an Fc variant that enhances FcγRIIb binding while distinguishing FcγRIIb and FcγRIIa^R131^ precisely.

To elucidate the structural mechanism by which P238D variant discriminates FcγRIIb from FcγRIIa^R131^, we solved the crystal structure of the complex of Fc(P238D) and FcγRIIb. Our structural analysis indicated that the high selectivity of P238D variant was achieved by a dynamic conformational change of the Fc-FcγRIIb interface that was induced by P238D substitution into wild-type IgG1. In reported Fc(IgG1) structures, Pro238 forms a hydrophobic core with its surrounding residues. Therefore, substituting this hydrophobic Pro238 to hydrophilic Asp should cause large free energy loss for desolvation of Asp to maintain the same structure. To avoid this free energy loss, Asp238 showed a large shift out of its original position to achieve access to the solvent region. Additionally, the position of Pro238 in Fc(IgG1) was occupied by Leu235 instead in C_H_2-A of Fc(P238D) complexed with FcγRIIb. As the result, the large conformational change of loop233–240 attached to the hinge region was observed. This change would affect the domain arrangement between the C_H_2-A and B domains because both C_H_2 domains connect by S–S bonds in the hinge region and cannot move independently. In fact, the relative arrangement of C_H_2-A and B domains in the Fc(P238D)/FcγRIIb complex was different from that of Fc(IgG1)/FcγRIIa^R131^, despite FcγRIIb having the highest homology to FcγRIIa^R131^. As the result of these dynamic conformational changes, Fc(P238D) acquired two novel interactions with FcγRIIb. One is the hydrogen bond between the main chain of Gly237 in C_H_2-A and the side chain of Tyr160 in FcγRIIb. Both FcγRIIa allotype cannot make this hydrogen bond because corresponding residue of this Tyr is Phe in FcγRIIa (Supplementary Fig. S1). So, this interaction would play a critical role for distinguishing FcγRIIb from FcγRIIa. The other one is the salt bridge between Asp270 in C_H_2-A and Arg131 of FcγRIIb. This salt bridge would contribute to improve not only the binding affinity to FcγRIIb but also the selectivity over FcγRIIa^H131^ because this allotype has His as the corresponding residue of Arg131 in FcγRIIb. On the other hand, the S267E/L328F variant increased binding affinity to both FcγRIIb and FcγRIIa^R131^ to the same extent, while it did not increase binding affinity to FcγRIIa^H131^ or other FcγRs. From the reported complex structure of Fc(IgG1)/FcγRIIa^R131^, we elucidated that Glu267 of the Fc with S267E/L328F substitution would form a salt bridge with Arg131 of the FcγRIIb and also of the FcγRIIa^R131^. This might explain the reason for the lack of selectivity of the S267E/L328F variant to FcγRIIb over FcγRIIa^R131^, both of which have Arg at position 131. Large conformational change induced by P238D would also explain the reason for lack of additive effect of combining P238D with L328E or S267E/L328F.

Then, in order to further increase the binding affinity to FcγRIIb, we conducted a second comprehensive mutagenesis using the P238D variant as a template rather than combining substitution(s) effective for wild-type IgG1 template, since the interface of Fc(P238D)/FcγRIIb was considered to be significantly changed from that of Fc(IgG1)/FcγRIIb. P238D-based comprehensive and combinatorial study identified V12 variant whose affinity for FcγRIIb was significantly increased from P238D variant. Especially, P271G markedly increased affinity for FcγRIIb. The substitution of fixed proline to flexible glycine would contribute to release conformational stress when a salt bridge between Asp270 in Fc (V12) and Arg131 in FcγRIIb is formed, which is considered to contribute to the affinity improvement for FcγRIIb.

Structural analysis of P238D suggests that P238D substitution seemed to destabilize the hydrophobic core of C_H_2 domain, although this could be partially compensated by Leu235 as found in C_H_2-A domain. The conformational changes seem to be the cause of decreased *T*_M_ for V12 variant. Although this reduced *T*_M_ raised concern regarding the storage stability of Fc(V12) for pharmaceutical application, its stability (aggregation tendency at 100 mg/ml) was comparable with that of wild-type IgG1.

It is known that, for IgG1 antibody to have a long half-life *in vivo*, binding to FcRn at acidic pH is important. The affinity of V12 variant for hFcRn was comparable with that of wild-type IgG1 and, consistently, the *in vivo* half-life of V12 variant was also comparable with that of wild-type IgG1. However, it should be noted that because the binding affinity of V12 variant to murine FcγRIIb (the mouse counterpart of human FcγRIIb) was not increased, its effect on the pharmacokinetics was not addressed in this study.

ICs induce platelet aggregation and activation when antibody binds to FcγRIIa expressed on the platelet surface (Boumpas *et al.*, 2003; Scappaticci *et al.*, 2007). The previous studies suggest that ICs consisting of IgG with enhanced binding to FcγRIIa have the potential to induce platelet aggregation and activation more intensively than IC consisting of wild-type IgG. However, the effect of engineered Fc with enhanced binding affinity to FcγRIIa on platelet activation and aggregation induced by IC has not so far been investigated. In this report, IC consisting of IgE and anti-IgE antibody with enhanced binding affinity to both FcγRIIb and FcγRIIa^R131^ (S267E/L328F variant) induced the activation and aggregation of platelets obtained from FcγRIIa genotype of R/R131 homozygous donors, but not from FcγRIIa genotype of H/H131 homozygous donors. On the other hand, anti-IgE antibody with selectively enhanced binding affinity to FcγRIIb did not induce the activation or aggregation of platelets from donors with either genotype. Platelets whose FcγRIIa genotype is R/H131 are considered to express both FcγRIIa allotypes on their surface, while platelets whose FcγRIIa genotype is R/R131 express only FcγRIIa^R131^. Therefore, a similar tendency will be observed in the platelets from heterozygous donors, although the induction of the aggregation and activation might be milder.

It is known that FcγRIIa internalizes ICs by endocytosis and transfers them to lysosomal degradation (Zhang and Booth, 2010), while FcγRIIb recycles the internalized ICs (Bergtold *et al.*, 2005; Mousavi *et al.*, 2007). These reports suggest that an antibody with enhanced binding affinity to FcγRIIa would be more rapidly eliminated from plasma by FcγRIIa-mediated uptake and degradation than wild-type IgG1, while an antibody with enhanced binding only to FcγRIIb would have a longer half-life because the antibody would be recycled back to the cell surface. Indeed, an antibody with enhanced binding to both murine FcγRII and FcγRIII (counterpart of human FcγRIIa) exhibited more rapid clearance from plasma compared with an antibody with enhanced binding only to murine FcγRII (data not shown).

These facts indicate that therapeutic IgG with S267E/L328F substitution has the potential to induce platelet aggregation and activation and to be rapidly cleared from plasma in patients with FcγRIIa^R131^ genotype. One report described that the allelic frequency of R/R131, R/H131 and H/H131 is 31, 44 and 25%, respectively, among healthy Caucasians, and 26, 43 and 31%, respectively, among healthy African Americans (Lehrnbecher *et al.*, 1999). Another report showed that in Japanese, Chinese and Asian Indian populations the R/R131 genotype occurs in 6, 6 and 31%, respectively (Osborne *et al.*, 1994). Considering the proportion of populations with R/R and R/H genotypes, enhancing the binding affinity to FcγRIIa^R131^ would have a substantial impact.

Previous studies using S267E/L328F or S267E substitution(s) have demonstrated that enhancing affinity to FcγRIIb is a promising application for therapeutic antibodies against CD19, IgE, DR5 and CD40 (Li and Ravetch, 2011, 2012; Chu *et al.*, 2012; Hammer, 2012). Consistent with these reports, V12 variant, as well as S267E/L328F variant, enhanced agonistic activity of antibody against CD137, one of TNFR superfamily molecules, compared with intact human IgG1. Since agonistic antibodies against TNFR superfamily are currently being explored for cancer immunotherapy, enhancement of the agonistic activity of these antibodies by selectively improving the binding affinity for FcγRIIb could be a promising approach. In addition, in Ba/F3 cells expressing constitutively active mutants of the receptor tyrosine kinase, Kit, ICs that crosslinked FcγRIIb and Kit inhibited growth factor-independent proliferation (Malbec and Daëron, 2012). In another report, ICs suppressed the TLR4-mediated response of DCs in rheumatoid arthritis patients through FcγRIIb. Each of these effects of FcγRIIb could be enhanced by applying Fc with enhanced affinity for FcγRIIb. Moreover, ICs significantly suppressed expression of CD40, CD80 and CD86 on FcγRIIb-overexpressing DCs, suggesting that in DCs, using ICs consisting of an antibody variant with selectively enhanced FcγRIIb affinity relative to FcγRIIa might polarize IC-triggered activating signals to inhibitory signals (Zhang *et al.*, 2011).

## Conclusion

In this study, we screened antibody Fc variant which selectively enhances the binding affinity to FcγRIIb over both FcγRIIa^R131^ and FcγRIIa^H131^ by comprehensive mutagenesis. We identified a distinct substitution, P238D, that could discriminate FcγRIIb from FcγRIIa^R131^ precisely, and crystal structural analysis revealed that this substitution substantially changed the recognition interface of Fc-FcγRIIb. We further designed an antibody variant with 200-fold higher affinity for FcγRIIb than IgG1 without increasing the affinity for other active FcγRs. The variant was comparable with IgG1 in terms of pharmacokinetics and storage stability. We also showed that an antibody with increased affinity for FcγRIIa has an increased possibility of inducing platelet activation and aggregation and of being rapidly cleared from plasma. Since previous studies and our study using agonist anti-CD137 antibody suggested that increasing the binding affinity to FcγRIIb has various therapeutic applications, our engineered Fc, which enhances binding selectivity to FcγRIIb, is expected to have a significant therapeutic potential.

## Supplementary data

Supplementary data are available at *PEDS* online.

## Funding

This work was fully supported by Chugai Pharmaceutical Co., Ltd. Funding to pay the Open Access publication charges for this article was provided by Chugai Pharmaceutical Co., Ltd.

## Supplementary Material


