# Correction to: Engineered antibody Fc variant with selectively enhanced FcγRIIb binding over both FcγRIIa^R131^ and FcγRIIa^H131^

**DOI:** 10.1093/protein/gzag018

**Published:** 2026-07-15

This is a correction to: F. Mimoto, H. Katada, S. Kadono, T. Igawa, T. Kuramochi, M. Muraoka, Y. Wada, K. Haraya, T. Miyazaki, K. Hattori, Engineered antibody Fc variant with selectively enhanced FcγRIIb binding over both FcγRIIa^R131^ and FcγRIIa^H131^, *Protein Engineering, Design and Selection*, Volume 26, Issue 10, October 2013, Pages 589–598, https://doi.org/10.1093/protein/gzt022

In the originally published version of this manuscript, the supplementary data files were incomplete.

The omitted files have been added in this correction notice.

These details have been corrected only in this correction notice to preserve the version of record.

## Supplementary data

Supplementary data are available at *PEDS* online.

