# Supplementary material for: Engineered antibody Fc variant with selectively enhanced FcγRIIb binding over both FcγRIIaR131 and FcγRIIaH131
Source: Protein Eng Des Sel. 2013 Jun 5;26(10):589–98. doi: 10.1093/protein/gzt022 (PMC3785249; doi:10.1093/protein/gzt022)
Supplement: Supplementary file 1 [file gzt022-gzt022supp.doc]

**Supplementary material for Engineered antibody Fc variant with selectively enhanced FcγRIIb binding over both FcγRIIa^R131^ and FcγRIIa^H131^**

Authors

F. Mimoto, H. Katada, S. Kadono, T. Igawa*, T. Kuramochi, M. Muraoka, Y. Wada, R. Saito, K. Haraya, T. Miyazaki, K. Hattori

Chugai Pharmaceutical Co. Ltd., Research Division

**Corresponding author:*

Tomoyuki Igawa

TEL: +81(550)87-3029; FAX: +81(550)87-5326

E-mail address: igawatmy@chugai-pharm.co.jp

**Supplementary materials and methods**

*P-selectin and PAC-1 expression*

Washed platelets were incubated with preformed IC for 5 min at room temperature and activated by 30 µM ADP. The platelets were stained by a phycoerythrin-conjugated anti-human P-slectin antibody (BECTON DICKINSON), a peridinin chlorophyll protein complex-conjugated anti-CD61 antibody (BD Biosciences) or FITC-conjugated anti-PAC-1 antibody (BD Biosciences). P-selectin and PAC-1 expression was analyzed using a flow cytometer (FACS CantoII, BD Biosciences).

**Supplementary figures**

Fig. S1

Sequence alignment of extracellular 2^nd^ domain of human FcγRIIb, FcγRIIa^R131^, FcγRIIa^H131^.

Residues identical to the sequence of FcγRIIb are shown by (. . . ) and the previously reported interaction site for the Fc region is framed (Ramsland *et al.*, 2011). An arrow indicates the residue at position 131 or 160.

Fig. S2

The expression levels of CD62p and Pac-1 on platelets incubated with ICs. Expression of CD62p and Pac-1 was evaluated by FACS analysis after platelets were incubated with ICs consisting of IgE with anti-IgE V12 variant (blue), that of anti-IgE S267E/L328F variant (red), IgE and anti-IgE IgG1 (green), or PBS (black) for 5 min before being primed with ADP. The expression profiles of CD62p and Pac-1 of the platelets from two donors with FcγRIIa R/R131 homozygous genotype are shown in (A) and (B), respectively. The expression profiles of the platelets from two donors with FcγRIIa H/H131 homozygous genotype are shown in (C) and (D), respectively.
